# A fully caninised anti-NGF monoclonal antibody for pain relief in dogs

**DOI:** 10.1186/1746-6148-9-226

**Published:** 2013-11-09

**Authors:** David P Gearing, Elena R Virtue, Robert P Gearing, Alexander C Drew

**Affiliations:** 1 Nexvet Biopharma Pty Ltd, Level 39, 385 Bourke St, Melbourne, Victoria 3000, Australia; 2 Centre For Innate Immunity & Infectious Diseases, Monash Institute Of Medical Research, Monash University, Clayton, Victoria 3168, Australia

**Keywords:** Nerve growth factor, Analgesia, Companion animals, Monoclonal antibody, Pharmacokinetics, Chronic pain, Veterinary bio-therapeutic

## Abstract

**Background:**

Monoclonal antibodies are a major class of biological therapies in human medicine but have not yet been successfully applied to veterinary species. We have developed a novel approach, PETisation, to rapidly convert antibodies for use in veterinary species. As an example, anti-nerve growth factor (anti-NGF) monoclonal antibodies (mAbs) which are effective in reducing acute and chronic pain in rodents and man are potentially useful for treating pain in dogs but a fully caninised mAb is required in order to avoid an immune response. The aim of this study was to determine the optimal properties of a caninised anti-NGF mAb for safe, repeated administration to dogs, to determine its pharmacokinetic properties and to evaluate its efficacy in a model of inflammatory pain in vivo.

**Results:**

Starting with a rat anti-NGF mAb, we used a novel algorithm based on expressed canine immunoglobulin sequences to design and characterise recombinant caninised anti-NGF mAbs. Construction with only 2 of the 4 canine IgG heavy chain isotypes (A and D) resulted in stable antibodies which bound and inhibited NGF with high-affinity and potency but did not bind complement C1q or the high-affinity Fc receptor gamma R1 (CD64). One of the mAbs (NV-01) was selected for scale-up manufacture, purification and pre-clinical evaluation. When administered to dogs, NV-01 was well tolerated, had a long serum half-life of 9 days, was not overtly immunogenic following repeated dosing in the dog and reduced signs of lameness in a kaolin model of inflammatory pain.

**Conclusions:**

The combination of stability, high affinity and potency, no effector activity and long half-life, combined with safety and activity in the model of inflammatory pain in vivo suggests that further development of the caninised anti-NGF mAb NV-01 as a therapeutic agent for the treatment of chronic pain in dogs is warranted.

## Background

Current therapeutic options for pain management in dogs are limited to a few classes of drugs including non-steroidal anti-inflammatory drugs (NSAID), narcotics and polysulphated glycosaminoglycans (PSGAG) [1,2]. Alternative therapeutic options are desirable, in particular for the management of chronic pain. Recently, a new class of antibody drugs have been developed which provide effective analgesia in rodents and man through interference of binding of NGF to its cellular receptors on nociceptive neurons.

Whereas during mammalian development, NGF is essential for the survival of sensory and sympathetic neurons [3,4] in the adult it is expressed locally at sites of injury and inflammation and is a major factor promoting pain and hyperalgesia [5,6]. NGF is produced by a variety of inflammatory and immune cells, joint chondrocytes and has also been detected in nerve and neuroma preparations [5]–[7]. Following binding to its receptor trkA on nociceptors, NGF causes immediate and long-termexcitability through activation of ion channels, the transient receptor potential vanilloid receptor (TRPV1) and secondary neurotransmitters including substance P and brain-derived neurotrophic factor (BDNF) [5]–[7]. NGF also causes the sprouting of nerve endings into the site of inflammation but does not appear to play a role in inflammation *per se*[8]. Furthermore, mutations in NGF and its trkA receptor are associated with diminished pain responses [5]–[7]. Neutralising antibodies to NGF are highly effective analgesics in rodent models of inflammatory pain, arthritis pain, cancer pain, and bone fracture pain [5]–[7]. This encouraging biological activity has resulted in the development of several NGF antagonists for the treatment of pain in man.

The clinical efficacy of anti-human NGF mAbs has been demonstrated in several human studies including several large-scale phase 3 clinical trials: responses to the pain associated with osteoarthritis (OA), lower back pain and cystitis have been evaluated [5]–[7,9]–[12]. The anti-NGF antibodies were generally very well tolerated (consistent with a benign profile in 6-month primate studies [13]), with mild to moderate, transient peripheral sensation changes as the only consequences [7]. A small number (16 of 6,800) of patients with OA that were treated with anti-NGF mAbs required joint replacement earlier than would be normally expected [14] and this was attributed to “rapidly progressing osteoarthritis”. The cause of this worsening has been debated, although in some patients, the accelerated osteoarthritis was possibly due to concomitant NSAID use [15].

Canine NGF and its receptor are closely homologous to those of other species. NGF and trkA are expressed in similar tissues in dog and man, appear to be under similar control mechanisms, and have similar functions [16]–[19]. NGF levels are significantly elevated in the synovial fluid of osteoarthritic dogs with chronic lameness [20].

As with other mammals, the canine immune system shares major immunoglobulin types, including IgG (of which there are four isotypes: [21]) and IgG-Fc receptors including the high-affinity FcR CD64 [NCBI reference: NP_001002976.1, XP_003640260.1, XP_536141.3] [22] and the neonatal FcRn [XP_533618.2, XP_003640095.1], which potentiates IgG half-life *in vivo*[23,24].

Based on the conservation of the NGF system between dog and man, it was at least possible that the rat anti-human NGF MAb may also be reactive with canine NGF. Furthermore, if this reactivity was of high affinity which could be retained during the process of conversion to a fully caninised antibody, we postulated that the resulting antibody could well be an effective treatment for pain in the dog. We therefore converted a high-affinity, potent rat anti-NGF monoclonal antibody (αD11; [25]) to a fully canine form (NV-01) with no loss of bioactivity by a novel process we term "PETisation". Unlike other approaches for converting antibody sequences from one species for use in another, such as CDR grafting, which rely on germline sequences to provide recipient V domain framework structures, the PETisation approach makes use of sequences from expressed and circulating IgG. This increases the likelihood that the resulting antibody will be both active and recognized as self by the recipient species, thereby overcoming immunogenicity concerns.

The design, preparation and *in* vitro characteristics of NV-01, together with preliminary studies investigating its safety and effectiveness are described herein. Collectively they show that NV-01 is a potent inhibitor of NGF, is well tolerated and non-immunogenic and shows promise as an analgesic in dogs. These preliminary data support our hypothesis that NV-01 might be useful as a treatment for pain in dogs (e.g. treatment of joint pain associated with osteoarthritis, cancer pain and post-surgical pain) and suggest that its further development as a veterinary medicine is warranted.

## Methods

### Sources of NGF

**Figure 1:**
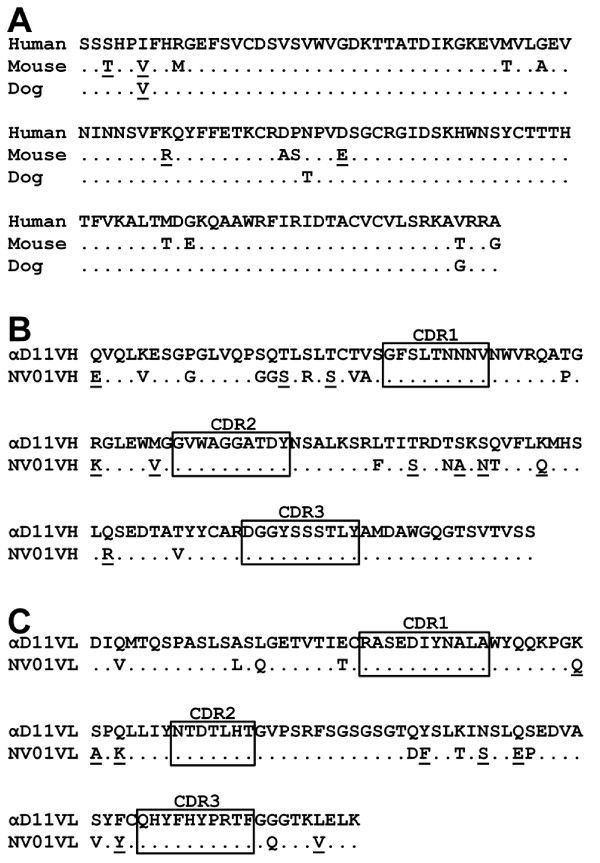
**NGF and anti-NGF antibody sequences. A)** Alignment of the mature peptide sequence of NGF from human, mouse & dog. Identical amino acids are indicated by dots and similar amino acids are underlined. **B)** Variable heavy &**C)** variable light chain sequences of the anti-NGF antibody *α*D11 aligned to the caninised antibody NV-01. Identical amino acids are indicated by dots and similar amino acids are underlined. Complementarity-determining regions (CDR) are boxed.

A cDNA sequence encoding the amino acid sequence of canine pre-pro beta NGF (Figure 1A) with a C-terminal poly-His tag was synthesized from oligonucleotides, cloned into pcDNA3.1+ expression vector and transiently transfected into HEK293 cells at Geneart AG (Life Technologies, Regensberg, Germany). The supernatant was harvested and purified by Ni-HiTrap chromatography (GE Healthcare, Upsalla, Sweden). Purified mouse NGF (muNGF) was purchased from Biosensis (Thebarton, Australia).

### Conversion of αD11 variable domains for use in the dog

In order to reduce the immunogenic potential of rat αD11 [25] in the dog, changes were made to the heavy and light chain variable domain framework sequences by alignment with a matrix of predicted protein sequences encoded by expressed canine IgG cDNA sequences. Where the αD11 sequence corresponded to the matrix, no changes were made. Where they differed, the most similar amino acid (by charge, size, polarity) in the matrix was substituted. If no similar amino acid was available, the most abundant canine residue was chosen. The changes made are illustrated in Figure 1B and 1C. Twenty-two substitutions were made to the heavy chain variable domain, of which 10 were conservative and 17 substitutions were made to the light chain variable domain, of which 9 were conservative. By this process, termed PETisation, the αD11 framework sequences were completely caninised, with minimal changes made from the donor αD11 antibody.

### Construction of NV-01 antibody heavy and light chains

The caninised αD11 heavy chain variable domain sequence (caN) was combined with the αD11 heavy chain signal sequence and the constant domain sequences of each of the four canine IgG heavy chain isotypes A,B, C and D [21] to form caN-HCA, caN-HCB, caN-HCC and caN-HCD sequences, respectively. The caninised NV-01 light chain variable domain sequence was combined with the αD11 light chain signal sequence and the constant domain sequence of the canine kappa light chain to form the caN-kLC sequence. The resulting amino acid sequences were converted to codon-optimized nucleotide sequences for expression in CHO cells (Geneart AG, Life Technologies, Regensberg, Germany) and these cDNA were cloned separately into pcDNA 3.1 + .

Co-expression in CHO cells by transient transfection of caN-kLC light chain cDNA with each of the four NV-01 heavy chain isotype cDNAs, caN-HCA, caN-HCB, caN-HCC and caN-HCD, produced supernatants containing antibodies “caN-HCA1 + kLC1”, “caN-HCB2 + kLC1”, “caN-HCC1 + kLC1” and “caN-HCD2 + kLC1”, respectively.

Further modification of the heavy chain framework sequence based on alignment to a larger number of canine cDNA sequences resulted in the modified heavy chain sequence “caN2-HCA1” (Figure 1). Co-expression of this sequence with light chain caN-kLC1 formed the antibody “caN2-HCA1-kLC1”, which was assigned the compound number NV-01.

### Recombinant antibody preparations

For small scale, antibodies were transiently expressed in CHO cells (Geneart AG, Life Technologies, Regensberg, Germany). The type B anti-NGF antibody (caN-HCB2 + kLC1) was purified on protein A from CHO cell supernatant. The type C anti-NGF antibody (caN-HCC1 + kLC1) was purified on Protein G from CHO cell supernatants. Type A & type D anti-NGF antibodies (caN-HCA1 + kLC1 and ca-HCD2 + kLC1) could not be purified using Protein A or G and were purified using Protein L.

For *in vivo* experiments, NV-01 antibody was expressed in CHO cells (Lonza Biologics plc, Cambridge, UK). Stable pooled transfections of CHO cells with cDNA encoding NV-01 heavy & light chains were cultured in a fed batch system for 13 days, before harvesting of supernatant containing NV-01. Clarified supernatant was diluted 1:2 with 50 mM Tris pH 8.0. The protein was captured on a HiTrap 5 ml anion exchange Q FF column (GE Healthcare) and impurities removed by washing the column with 50 mM Tris, 100 mM NaCl, pH 8.0. The protein was eluted with 50 mM Tris, 200 mM NaCl, pH 8.0.

Anion exchange fractions containing antibody were concentrated and diluted 1:10 with 50 mM sodium phosphate, 1 M ammonium sulphate, pH 7.0. The protein was captured on a HiTrap hydrophobic interaction Phenyl HP column (GE Healthcare) and impurities removed by washing the column with 50 mM sodium phosphate, 1 M ammonium sulphate, pH 7.0 (loading buffer). The protein was eluted with a linear gradient from loading buffer to 50 mM sodium phosphate, pH 7.0.

Material from the hydrophobic interaction step was further purified by size exclusion chromatography (HiLoad Superdex 200 pg 16/60, GE Healthcare), then concentrated and formulated into phosphate buffered saline (PBS) pH 7.3 by ultrafiltration (Amicon Ultra-15, molecular weight cut-off 30,000; Millipore, Billerica, USA).

NV-01 produced by this method was determined to be >95% pure and 100% monomeric by size exclusion HPLC. The preparations were free of detectable endotoxin (<0.1 EU/mL; Endosafe®-PTS™ Charles River Laboratories, Wilmington, USA).

### Anti-NGF antibody detection by ELISA

ELISA plates were coated with 0.1 μg/ml muNGF and blocked with 5% BSA/PBS. muNGF coated wells were incubated for 1 h at room temperature with recombinant canine anti-NGF IgG preparations, diluted in PBS/1% BSA. Antibody concentrations ranging from 40 ng/ml to 0.625 ng/ml were used to establish a standard curve. After washing, the plates were incubated with a 1/5000 dilution of rabbit anti-canine IgG-HRP (Sigma, St. Louis, USA) in PBS/1% BSA. Plates were washed with PBS 0.05% Tween 20 and developed by the addition of TMB substrate (Thermo Scientific, Waltham, USA). Development was stopped by the addition of 2 M H_2_SO_4_ and absorbance read at 450 nm and background was subtracted.

For the detection of NV-01 in canine plasma samples, the canine plasma was diluted and used in the ELISA as above. The background for the canine plasma was determined from the O.D. 450 nm of time zero serum.

### Complement C1q binding ELISA

NV-01 cDNA transfected CHO cell supernatants were concentrated using Vivaspin 20 concentrators (30 kDa cut-off, Vivaproducts, Littleton, USA). The concentrations of recombinant antibodies in the CHO cell supernatants and of purified antibodies were determined by titration in the anti-NGF ELISA, by comparison with standard preparations of purified B isotype canine anti-NGF antibody (caN-HCB2 + kLC1) or NV-01.

C1q binding was assayed following the method of Lewis et al. [26]. Plates were coated with 2.5 μg/ml muNGF and blocked with 5% BSA/PBS. Coated wells were incubated for 1 h at room temperature with recombinant canine anti-NGF IgG, diluted in PBS/1% BSA. Antibody concentrations ranged from 10 μg/ml to 0.5 μg/ml. The plates were washed and incubated for 1 hour at room temperature with human serum or heat inactivated human serum diluted 1/100 in veronal buffered saline containing 0.05% Tween-20, 0.1% gelatine and 0.5% BSA. After washing, plates were incubated with a 1/800 dilution of sheep anti-human C1q-HRP (AbD Serotec, Kidlington, UK) in PBS/1% BSA. After washing, plates were developed by the addition of TMB substrate (Thermo Scientific). Development was stopped by the addition of 2 M H_2_SO_4_ and absorbance read at 450 nm. The O.D. 450 nm obtained using heat-inactivated serum was used as background and subtracted from the O.D. 450 nm obtained using untreated serum.

### Soluble CD64 binding assay to caninised antibodies

The sequence of cDNA encoding canine CD64, the high affinity Fc receptor gamma R1, was obtained from Ensembl [http://www.ensembl.org: ENSCAFT00000018253]. The sequence was modified as previously used to generate a soluble human CD64 [27]. The trans-membrane domain was removed and c-myc and poly-His tags were added to the C-terminus to allow for detection and purification. The cDNAs were synthesised, cloned into expression vectors and protein was expressed by transient transfection of HEK suspension cells (Geneart AG, Life Technologies, Regensberg, Germany). Soluble canine CD64 (scaCD64) was purified from the HEK cell supernatants by Ni-Hi Trap chromatography and size exclusion chromatography (as above).

Microtitre plates were coated with 0.1 μg/ml muNGF and blocked with 5% BSA/PBS/0.05% Tween-20. Coated wells were incubated for 1 h at room temperature with recombinant canine anti-NGF IgG, diluted in PBS/1% BSA/0.05% Tween-20. Antibody concentrations ranged from 100 ng/ml to 1.6 ng/ml. After washing, plates were incubated with 1 μg/ml purified scaCD64. Plates were washed and binding of scaCD64 detected by the addition of anti-c-myc-HRP (Pierce) in PBS/1% BSA/0.05% Tween-20. After washing plates were developed by the addition of TMB substrate (Thermo Scientific). Development was stopped by the addition of 2 M H_2_SO_4_ and absorbance read at 450 nm. The background (no canine IgG) was subtracted.

### Antibody binding kinetics

The binding affinity of NV-01 to muNGF was analysed by Surface Plasmon Resonance (SPR) using a ProteOn XPR36 SPRi biosensor equipped with a GLM chip (BioRad, Hercules, USA). The chip was conditioned with 0.5% SDS, 50 mM NaOH and 100 mM HCl. Following conditioning, the lanes were activated using equal parts of EDAC and NHS amine coupling reagents (BioRad). The NGF protein was immobilised to the chip at a concentration of 50 μg/mL in sodium acetate buffer (pH 4.5). Following immobilisation all three channels were deactivated using ethanolamine. NV-01 was passed across the surface at 500 nM, 250 nM, 125 nM, 62.5 nM and 31.25 nM. The binding was displayed as a spectrogram. Controls were subtracted to give specific binding. A Langmuir curve fit model was then used to determine the specific affinity.

### TF-1 proliferation-inhibition assay

TF-1 cells were maintained in RPMI 1640 medium, supplemented with 10% foetal calf serum, 10 mM HEPES, penicillin/streptomycin (10 U/ml and 100 ug/ml final respectively) and 2 ng/ml GM-CSF. The TF-1 cells were centrifuged and resuspended in "starve media" (as above with no GM-CSF). The flasks were incubated in a humidified 37°C, 5% CO_2_ incubator for 24 hours. For the assay, 1x10^5^ GM-CSF starved cells were used per well. Half-maximal proliferation of TF-1 cells was observed at 1 ng/mL muNGF and dilutions of NV-01 (2.5 ng/ml to 0.002 ng/ml) were titrated against this concentration of NGF. The plates were incubated for 48 hours at 37°C/5% CO_2_ prior to measuring proliferation CellTiter 96 Aqueous One Solution Cell Proliferation Assay, Promega, Madison, USA). The assay was performed in triplicate. A mouse IgG2a mAb (eBM2a, eBioscience, San Diego, USA) was used as a negative control.

### Model of inflammatory pain

A model of inflammatory pain in the cat, induced by injection of kaolin into the footpad [28], was adapted for use in the dog ([29], following institutional ethics review; at Charles River Laboratories (CRL), Ballina, Ireland). Following kaolin injection into the footpad, the dog becomes lame in that leg within 24 h and then progressively recovers over a period of 7–14 days and is then returned to the colony. Following review and approval by the CRL institutional ethics committee (ref AUS001\12-003), the kaolin model was used to assess the potential for NV-01 delivered by intravenous (i.v.) or sub-cutaneous (s.c.) routes as a pain-relieving drug in the dog. The study was positively controlled using oral meloxicam, given as a single loading dose (0.2 mg/kg) 24 h post-kaolin, followed by six daily maintenance doses of 0.1 mg/kg. Vehicle (PBS) was administered i.v. as a negative control on day 0. All dogs were prepared for i.v. administration to maintain blinding during the observation period. NV-01 was given once as a single i.v. or s.c. dose of 0.2 mg/kg. This dose was selected based on the human anti-NGF antibody tanezumab as used in human clinical trials [11]. To maintain blinding, the administration of the test articles were performed by investigators separate to those who assessed lameness. The investigators involved in the lameness assessments were masked to the treatments administered in order to reduce bias due to subjectivity. The investigators involved in the administration of NV-01, meloxicam or vehicle control were not masked.

Thirty-two dogs (17 male and 15 female) were enrolled in the study and randomly allocated to four treatment groups (n = 8 per group). Animals assigned to Group 1 served as a negative control group and were treated with PBS administered by i.v. infusion. Animals assigned to Group 2 were treated with NV-01 administered by i.v. infusion at a dose of 0.2 mg/kg once on Study Day 0. Animals assigned to Group 3 were treated with NV-01 administered by s.c. injection at a dose of 0.2 mg/kg once on Study Day 0. Animals assigned to Group 4 were treated with meloxicam, administered daily by oral administration using the recommended dose of 0.2 mg/kg on Study Day 0 and 0.1 mg/kg daily from Study Day 1 to Study Day 7.

The animals underwent experimental induction of paw inflammation using kaolin (Sigma). The degree of lameness induced by kaolin was scored according to 4 levels, where a score of 0 was not lame (full weight bearing), 1 was slightly lame (not fully weight bearing but walking well), 2 was moderately lame (slightly weight bearing and not walking well) and 3 was severely lame (not weight bearing). All animals that were enrolled in the study reached the required lameness score (3) 24 hours after kaolin administration. Endpoint assessments were performed at the following times: prior to kaolin administration, pre-treatment on Study Day 0; and at the following times after dosing: +0.5 h, +2 h, +4 h, +6 h, +1 d, +2 d, +3 d, +4 d, +5 d, +6 d and +7 d. The lameness scores were un-blinded and average scores from test article treated dogs were compared to the placebo control as previously described [28,29]. Statistical analysis was performed using a non-parametric Mann–Whitney test (one-tailed).

## Results

### Antibody design and expression in CHO cells

The αD11 antibody [25] binds with high affinity to mouse and human NGF but binding to canine NGF had not been previously described. Given the level of sequence similarity between mouse, human and canine NGF protein sequences (Figure 1A) we reasoned that αD11-based caninised antibodies might also bind canine NGF.

**Figure 2:**
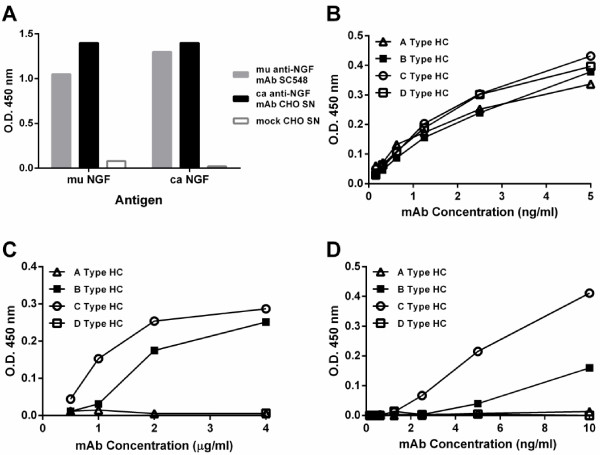
**Functional assays of caninised anti-NGF recombinant antibodies with different heavy chain (HC) isotypes. A)** Binding of canine anti-NGF antibody to murine (mu) and canine (ca) NGF. CHO cell supernatant containing canine anti-NGF B type heavy chain was compared with a mouse anti-NGF mAb (SC548) and mock transfected CHO cell supernatant. **B)** Binding to muNGF determined by ELISA. **C)** Complement C1q binding to antigen-captured antibodies detected by ELISA. **D)** High affinity Fc receptor binding. Soluble canine CD64 binding to antigen-captured antibodies detected by ELISA.

A small quantity of canine NGF with a C-terminal poly-His tag was synthesized in HEK-293 cells and purified by Ni-HiTrap chromatography. Supernatant of CHO cells expressing caN-HCB2 + kLC1 (see below) bound equally well to canine and mouse NGF by ELISA, when compared to a commercially available mouse anti-NGF antibody preparation (Figure 2A). The low yield of synthetic canine NGF precluded its further use and mouse NGF was used subsequently.

Co-expression in CHO cells of caN-kLC light chain cDNA with each of the four heavy chain isotype cDNAs caN-HCA, caN-HCB, caN-HCC and caN-HCD, produced supernatants containing antibodies “caN-HCA1 + kLC1”, “caN-HCB2 + kLC1”, “caN-HCC1 + kLC1” and “caN-HCD2 + kLC1”, respectively that bound to muNGF equally well by ELISA (Figure 2B).

### Complement C1q and CD64 binding by recombinant canine anti-NGF antibodies

Anti-NGF antibody supernatants were used in a complement C1q binding ELISA (Figure 2C). Wells incubated with supernatants containing A and D isotype heavy chains (caN-HCA + kLC and caN-HCD + kLC) showed no detectable binding of C1q. By contrast, C1q bound to wells incubated with supernatants containing B and C isotype heavy chain antibodies (caN-HCB-kLC and caN-HCC-kLC). These results indicate that antibodies constructed with canine isotype A and D type heavy chains are not likely to activate complement, whereas the B and C type heavy chains are likely to activate complement.

Each of the canine anti-NGF IgG isotypes was assayed for their ability to bind to the soluble canine CD64 (Figure 2D). Type A and D heavy chains (caN-HCA + kLC and caN-HCD + kLC) showed no binding, while types B and C (caN-HCB-kLC and caN-HCC-kLC) both bound the soluble form of CD64. The type C isotype had a higher level of binding compared to the type B isotype.

### Characterisation of purified NV-01

**Figure 3:**
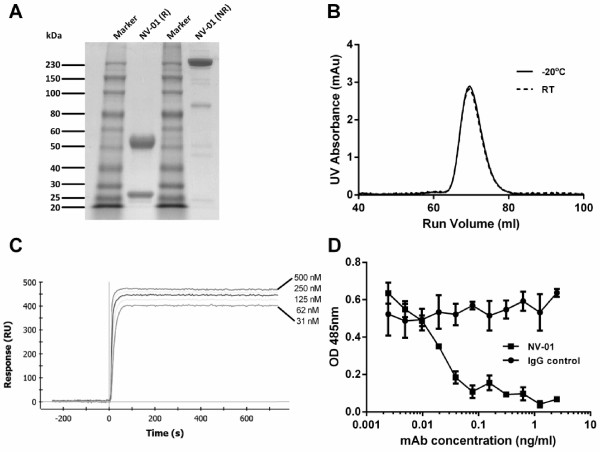
**Characterisation of the caninised anti-NGF antibody NV-01. A)** SDS-PAGE of reduced (R) and non-reduced (NR) NV-01. **B)** Stability of NV-01 determined by size-exclusion chromatography following storage at −20 degrees or room temperature (RT) for 3 months. **C)** Binding affinity of NV-01 to immobilized NGF. Various concentrations of NV-01 (500 nM, 250 nM, 125 nM, 62.5 nM and 31.25 nM) were passed over NGF for 60 seconds then the flow switched to buffer. **D)** Inhibition of NGF induced proliferation of TF-1 cells in vitro.1 ng/ml NGF was incubated with NV-01 or an irrelevant IgG control antibody at concentrations ranging from 2.5 ng/ml to 0.002 ng/ml.

Analysis by SDS-PAGE (Figure 3A) demonstrated the predicted heavy and light chain bands under reducing conditions and the predicted single band (two heavy chains and two light chains) under non-reducing conditions. A single peak was also observed by size-exclusion chromatography and this peak was stable to storage at −20°C and room temperature (~ 25°C) for 3 months (Figure 3B).

The binding affinity of NV-01 to NGF was assessed using biosensor assays (Figure 3C). Increasing concentrations of NV-01 were passed over immobilized NGF. After a 60 second binding phase, the flow was switched to buffer alone, and the near horizontal traces thereafter indicated a very low off rate. The binding affinity of NV-01 to NGF was determined to be <1 pM.

The human erythroleukaemic cell line, TF-1, proliferates in response to a number of cytokines including granulocyte-macrophage colony-stimulating factor (GM-CSF) and nerve growth factor (NGF) [30]. To determine the IC50 (dose that causes half-maximal inhibition) of NV-01, 1 ng/ml NGF was incubated with the NV-01 at concentrations ranging from 2.5 ng/ml to 0.002 ng/ml. The result was an IC50 of 0.02 ng/ml (Figure 3D).

### Safety, pharmacokinetics & immunogenicity of NV-01 in dogs

NV-01 was infused i.v. to 3 dogs at 2 mg/kg and the dogs were observed for 2 weeks. No adverse events were observed following infusion: there was no weight change over the following 14 days, no pyrexia, and standard clinical chemistry markers were within the standard range (data not shown).

**Figure 4:**
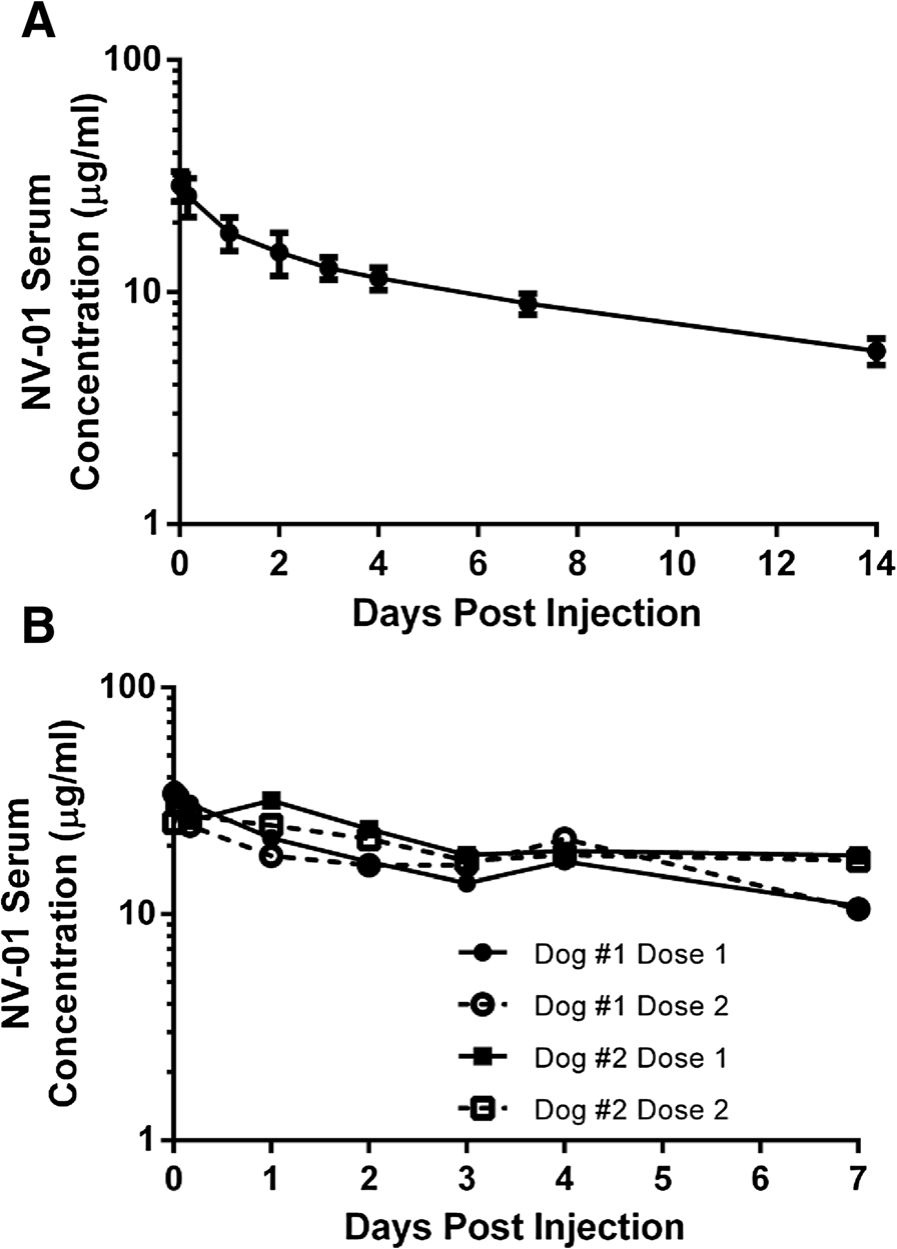
**Pharmacokinetic profile of NV-01 in the dog. A**) Single dose. NV-01 (2 mg/kg) was infused intravenously and the NV-01 serum concentration assayed by ELISA over a period of 14 days. **B)** Repeat dosing in two dogs. NV-01 (2 mg/kg) was administered by intravenous infusion twice, with a gap of eight months between each dose. The NV-01 concentration was determined by ELISA in serum samples taken over a period of 7 days after each infusion.

Plasma samples were taken at various times for subsequent pharmacokinetic (PK) analysis of NV-01 concentration using the NGF-binding ELISA. The PK profile from one dog, representative of the 3 dogs tested, is shown in Figure 4A. Analysis of the plasma levels of NV-01 over two weeks following injection demonstrated a two-phase PK profile typical of antibodies injected to other mammalian species, with a rapid tissue distribution (alpha) phase followed by a slower elimination (beta) phase (Figure 4A). The tissue distribution phase half-life was approximately12 hours and the elimination phase half-life was approximately 9 days (range 5–35 days, 95%CI).

Eight months later, two of the dogs were infused i.v. with a further dose of 2 mg/kg NV-01 and plasma samples were taken for PK analysis. PK analysis following the second dose of NV-01 gave a similar profile to that following the first dose (Figure 4B), indicating that NV-01 infusion did not induce an acute neutralising immunogenic response.

### Comparison of the efficacy of NV-01 and meloxicam in a model of inflammatory lameness in the dog

**Figure 5:**
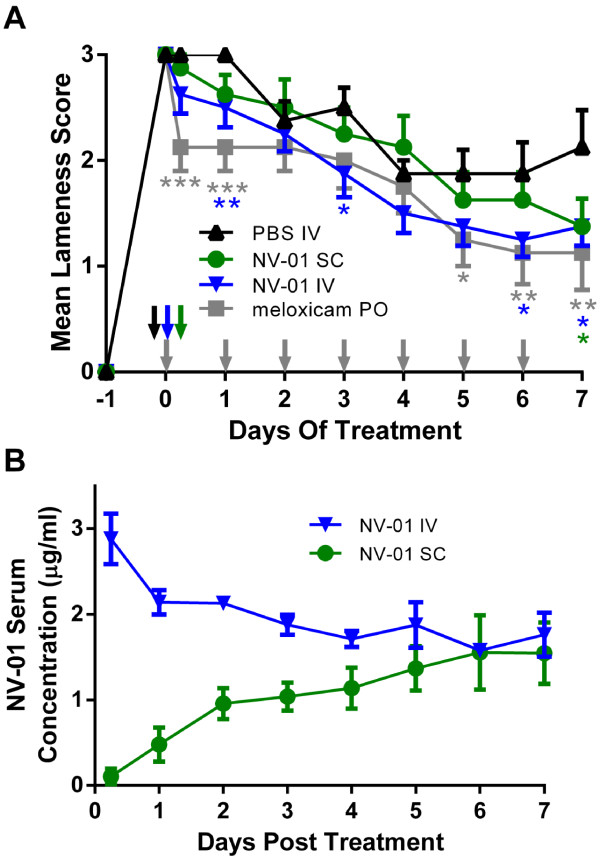
**Efficacy of NV-01 in a model of inflammation induced pain. A)** Average lameness scores are shown for each treatment group compared with the average lameness scores for the negative control group: The negative control was a single injection of phosphate buffered saline by intravenous infusion (IV, black triangles); Meloxicam (positive control) was given daily *per os* (PO) (grey squares); One 0.2 mg/kg dose of NV-01 was given by intravenous infusion (blue triangles); or one 0.2 mg/kg dose of NV-01 was given by subcutaneous injection (SC) (green circles). Points are the mean of eight animals per group. Error bars are the standard error of the mean. * p < 0.10; ** p < 0.05; *** p < 0.01 (Mann–Whitney test, one-tailed: test article compared to placebo). Arrows represent administration of the test article on the specified day. **B)** NV-01 serum concentration was determined by ELISA over a period of seven days in animals from the study given 0.2 mg/ml of NV-01 by intravenous infusion (blue triangles) or subcutaneous injection (green circles). Points are the mean NV-01 serum concentrations of three animals per group. Error bars are the standard deviation.

Kaolin injection to the footpad of dogs resulted in a consistent inflammatory lameness score of 3 (severely lame, not weight bearing) 24 hours later. Four groups of dogs were then treated with PBS placebo, oral meloxicam, i.v. NV-01 or s.c. NV-01. Lameness scores determined by assessors blinded to the type of intervention were determined on a four-point scale. Following 7 days of observations, the lameness scores were un-blinded, averaged according to intervention type and compared with placebo (i.v. PBS infusion). Figure 5A shows lameness scores for meloxicam-treated or NV-01-treated dogs compared with PBS-treated dogs.

Following the initial lameness caused by kaolin injection, the control PBS-injected dogs remained fully lame for another day, and then slowly but progressively improved over the following week (PBS IV, Figure 5A). As anticipated, daily oral meloxicam was effective in reducing lameness compared with i.v. PBS. The effect of daily oral meloxicam in reducing average lameness scores was matched by the single i.v. dose of NV-01: with meloxicam, significant difference (Mann–Whitney test: p ≤ 0.10) to placebo was observed at 6 h and on Day 1, 5, 6 and 7; whereas with i.v. NV-01, significance compared with placebo was seen on Day 1, 3, 6 and 7. (Figure 5A). A single s.c. injection of NV-01 was effective compared to placebo later with significance at Day 7. PK analysis of plasma NV-01 levels (Figure 5B) from the dogs treated with i.v. or s.c. NV-01 showed a delayed rise in circulating NV-01 following s.c. injection compared with i.v. infusion and that the circulating NV-01 levels were approximately equal 5–7 days following administration.

## Discussion

Methods for the conversion of antibodies from rodent to human are well described and are routinely used for the production of humanised antibodies for clinical use. For example CDR grafting [31,32] makes use of germline antibody framework sequence as donor sequence for primary antibody construction. This method often produces antibodies of lower affinity for antigen than the donor antibody. Affinity restoration or "maturation" is achieved by replacement of donor framework residues until affinity is restored but this often results in antibodies that are incompletely converted and so potentially more immunogenic. Rather than use germline donor sequences, we devised a simpler but more effective method (termed PETisation) using expressed cDNA sequences of the desired target species, in this case, the dog. Analysis of the greater range of framework sequence possibilities in canine IgG heavy and light chains enabled substitution of fewer (22 heavy chain) residues of the donor antibody to covert to a fully canine antibody framework than had we started with germline sequence (35 heavy chain). By making fewer changes, the resulting antibody is structurally most similar to the starting antibody and yet fully caninised. The advantages of this simple approach are: 1) the minimum changes are required to yield an antibody that has considerable similarity to the starting antibody, 2) the changes made are on the surface of the antibody structure and so do not affect the immunoglobulin fold and CDR presentation, and 3) the resulting antibody is completely caninised and so can be expected to be non-immunogenic.

The anti-NGF antibody αD11 is well described [7,25] and was considered a good candidate for conversion for use in dogs based on its high affinity to mouse and human NGF and its activity both *in vitro* and *in vivo*[7]. Although sequence differences between murine, human and canine NGF had the potential to affect αD11 binding, they were few and distant to the epitope [33]. Our results with NV-01 indicate that it retained the high affinity and activity of the αD11 antibody *in vitro* and was expressed at high levels in CHO cells and was stable following prolonged incubation at room temperature.

NGF is expressed on nerves and is subject to retrograde transport. The safe use of anti-NGF antibodies demands that the antibody not recruit effector functions of the immune system, such as complement-dependent cytotoxicity, and so avoid nerve damage. By direct analogy, anti-NGF antibodies for human use have been constructed from effector negative isotypes. The effector functions of canine antibodies were not previously described and so we expressed the caninised antibodies with each of the four canine isotypes previously described [21]. Two of the isotypes (IgG-heavy chain A and D) did not bind complement C1q (the first component of the complement cascade) and so were chosen for further development for their improved safety profile. Interestingly, neither antibody isotype bound to the high affinity FcR CD64 (a macrophage receptor involved in the processes of opsonisation and the respiratory burst), nor to Protein A or Protein G.

Anti-NGF mAb therapies are particularly attractive because of their potential to provide long-lasting analgesia in chronic disease settings, such as osteoarthritis and cancer pain. As a prelude to testing the potential of neutralising NGF in the management of pain in chronic canine diseases, a kaolin model of short -term inflammatory lameness was chosen to evaluate the efficacy of NV-01 in dogs in vivo. The kaolin model, first validated in cats and then in dogs, has the advantage that the animals recover quickly over the period of about 7–14 days and are returned to the colony. However, this ethical advantage also limits the model since there is not a stable period of inflammation during which direct comparisons can be made between drug and placebo [29]. Consequently, any drug benefit must be compared against a steady improvement in limb function in the placebo treated dogs. Presently, there are no ethically equivalent models of long-term inflammation in dogs and so the kaolin model was deemed most appropriate for this initial assessment of the potential pain-relieving properties of NV-01.

In the canine kaolin model, oral meloxicam therapy was effective at reducing signs of lameness in the first hours after treatment, whereas intravenously infused NV-01 required longer to become effective presumably due to slower distribution from the blood to inflamed tissue of the larger antibody molecule (Figure 5). Following this distribution phase, the single i.v. dose of NV-01 was similarly effective as daily oral meloxicam at reducing lameness. Sub-cutaneously delivered NV-01 was also shown to be active in reducing lameness, but somewhat later than following i.v. delivery due to the slower redistribution from skin to blood and then to inflamed tissue following the s.c. route of administration. Nonetheless, these studies confirm that both i.v. and s.c. routes of administration of NV-01 have potential to provide pain relief in dogs. As with studies of anti-NGF in the mouse [8], neither rectal temperature, nor paw circumference was affected by meloxicam or NV-01 therapies.

Compared with small molecule drugs, antibodies have long half-lives and are more suited to treatment of chronic diseases. Pharmacokinetic analyses showed that following i.v. infusion, NV-01 has an approximately 9-day half-life and has the potential to be administered repeatedly to dogs without inducing a neutralising immune response. The slow rate of decline shown in the pharmacokinetic analysis in Figure 4A suggests that a single injection may confer an extended period of therapeutic benefit in the treatment of chronic pain. This combination of safety, long half-life and low immunogenicity suggests that NV-01 should be further evaluated as a therapy for chronic pain conditions in dogs, where a safety profile superior to current therapies together with a long duration between injections would be clinically desirable attributes for a novel therapeutic agent.

## Conclusion

These preliminary studies lead us to conclude that the novel fully caninised anti-NGF mAb NV-01 shows considerable potential as an analgesic for dogs.

## Abbreviations

BSA: Bovine serum albumin; CDR: Complementarity-determining region; CHO: Chinese hamster ovary; EDAC: 1-ethyl-3-(3-dimethylaminopropyl) carbodiimide hydrochloride; ELISA: Enzyme-linked immuno-sorbent assay; FcR: Immunoglobulin Fc receptor; GM-CSF: Granulocyte colony-stimulating factor; HEK: Human embryonic kidney; i.v.: Intravenous; mAb: Monoclonal antibody; NGF: Nerve growth factor; NHS: N-hydroxysuccinimide; NSAID: Non-steroidal anti-inflammatory drug; PBS: Phosphate buffered saline; PK: Pharmacokinetic; s.c.: Subcutaneous; scaCD64: Soluble canine CD64; SDS-PAGE: Sodium dodecyl sulphate polyacrylamide gel electrophoresis.

## Competing interests

DG, RG and EV are employees and stockholders of Nexvet Biopharma Pty Ltd. DG has applied for patents relating to the content of the manuscript. AD is funded by a contract between Nexvet Biopharma and Monash University. Nexvet Biopharma is funding the article processing charges of this manuscript.

## Authors’ contributions

DG conceived the PETisation process, antibody design and *in vitro* assays, designed the *in vivo* studies and drafted the manuscript. EV carried out the cellular and biochemical assays. RG designed and coordinated the scale-up process. AD designed and carried out functional assays, immunoassays and protein purification. All authors read and approved the final manuscript.
